# The influence of pesticides on the corrosion of a Roman bowl excavated in Kent, UK

**DOI:** 10.1038/s41598-022-17902-9

**Published:** 2022-10-06

**Authors:** Luciana da Costa Carvalho, Dana Goodburn-Brown, James S. O. McCullagh, A. Mark Pollard

**Affiliations:** 1 School of Archaeology, University of Oxford, Oxford, OX1 3TG UK; 2 CSI: Sittingbourne, 22 The Forum, Sittingbourne, ME10 3DL UK; 3 Chemistry Research Laboratory, Department of Chemistry, University of Oxford, Oxford, OX1 3TA UK

**Keywords:** Analytical chemistry, Environmental impact

## Abstract

We analysed corrosion from a copper bowl dating from the Roman period (43–410 AD) found in a farm in Kent, UK. Despite its relatively good condition, the interior and exterior surface of the object had areas of deterioration containing green and brown-coloured corrosion which were sampled for characterization by a multi-analytical protocol. Basic copper chlorides atacamite and paratacamite were identified in the context of mineral phases along with chlorobenzenes in the green corrosion. Chlorobenzenes are common soil contaminants in rural areas from the use of pesticides, many of which were banned more than 50 years ago. Here we show that their presence is associated with accelerated corrosion, and this provides a threat to the preservation of archaeological metal objects in the ground.

## Introduction

**Figure 1:**
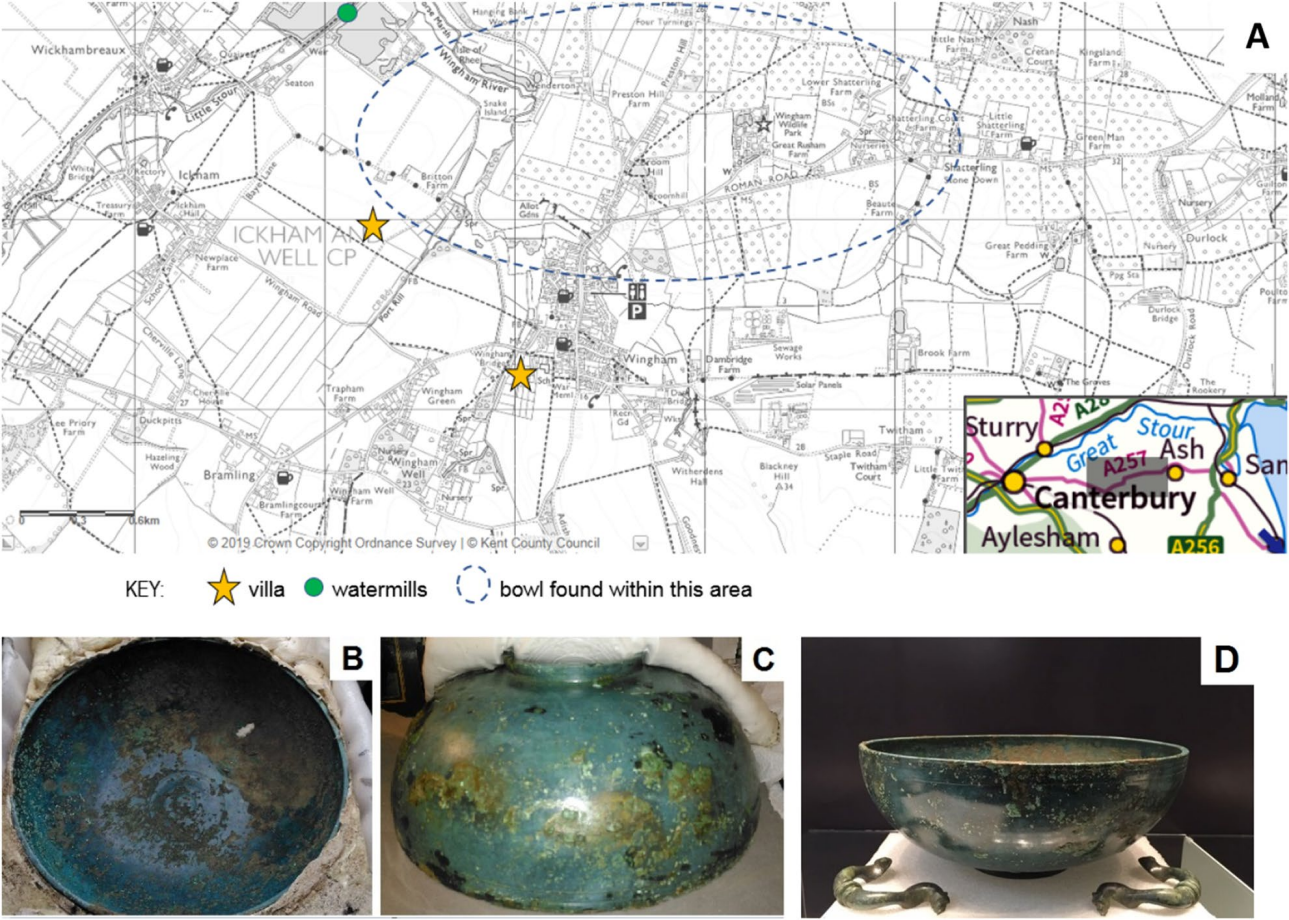
The recent history of the Roman Bowl–(**A**) the area where the bowl was found in relation to other Roman sites, exact findspot cannot be shown to protect the site (map created by Luciana da Costa Carvalho using https://webapps.kent.gov.uk/KCC.HeritageMaps.Web.Sites.Public/Default.aspx; (**B**) the interior and (**C**) exterior of the bowl during conservation and (**D**) the bowl on display at Sandwich Museum.

In November 2016, a metal detectorist discovered a copper bowl (Fig. 1A) whilst scanning a grassy track in an orchard at a farm near Wingham in Kent, UK. After the discovery, the bowl was left protected in situ so that it could be excavated a month later by a team from Canterbury Archaeological Trust and Dover Archaeological Group. A 2-m area around the bowl was exposed but no cremation or burial deposits associated with the it were found. The archaeologists block-lifted the objects in its surrounding soil and transported it to the CSI: Sittingbourne laboratory^1^ for conservation (Figs. 1B,C) in preparation for its display at Sandwich Museum (Fig. 1D).

The bowl was found mid-way between the important Roman settlements at Richborough and Canterbury, close to two large Roman villas and Roman watermills^2^.

It was found buried at a depth of 40 cm in a clay soil with chalk inclusions. Features revealed during the excavation indicate that the bowl was deliberately placed in a ditch within a settlement site previously unknown to archaeology. Pottery sherds and coins found in the site indicate that it originated in the Late Iron Age, with continued occupation into the later Roman times^1^.

During conservation, samples of the brown and green corrosion from the interior and exterior surface of the bowl were collected for analysis by a protocol combining different analytical techniques developed to target organic residues trapped in copper corrosion^3^ comprising of:

- X-ray powder Diffraction (XRD) for the identification of mineral phases;
- Fourier Transform Infrared (FTIR) for the identification of the chemical fingerprint;
- Gas Chromatography with quadrupole time-of-flight mass spectrometry (GC-QTOF-MS) with a thermal separation probe (TSP) for recovery and identification of organic molecules.

## Results

### Basic copper chlorides identified as mineral phases in the green corrosion

**Figure 2:**
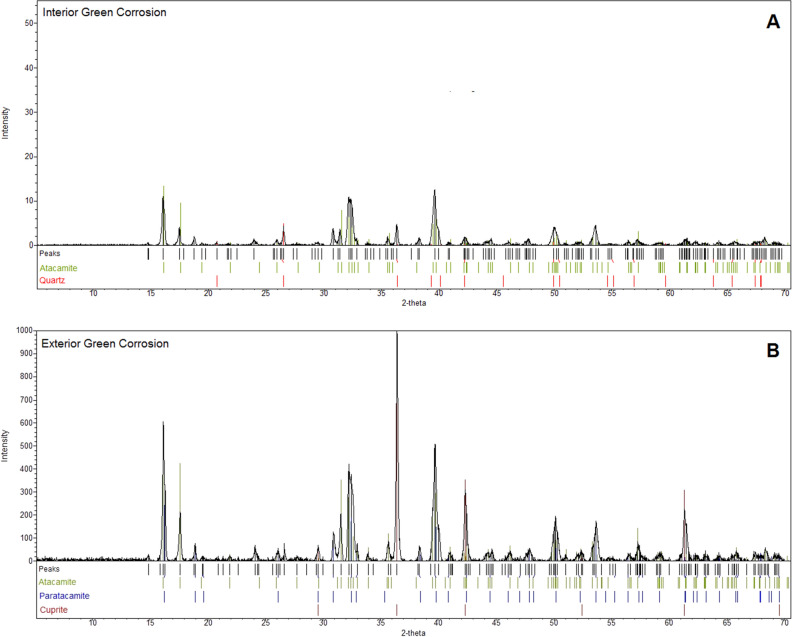
Diffractogram from the XRD analysis of the green corrosion material from the interior of the bowl (**A**) and the exterior (**B**), both showing identified mineral phases.

The mineral phases identified in the green corrosion from the interior of the bowl (Fig. 2A) were atacamite Cu_2_Cl(OH)_3_ and quartz. Atacamite was also identified as the main mineral phase in the exterior green corrosion (Fig. 2B), which also contained paratacamite Cu_3_(OH)_6_Cl_2_ and cuprite Cu_2_O. Atacamite and paratacamite are basic copper chlorides which interconvert^4^, and are typical copper corrosion products formed under acidic conditions and in an environment rich in chloride ions^5^.

**Figure 3:**
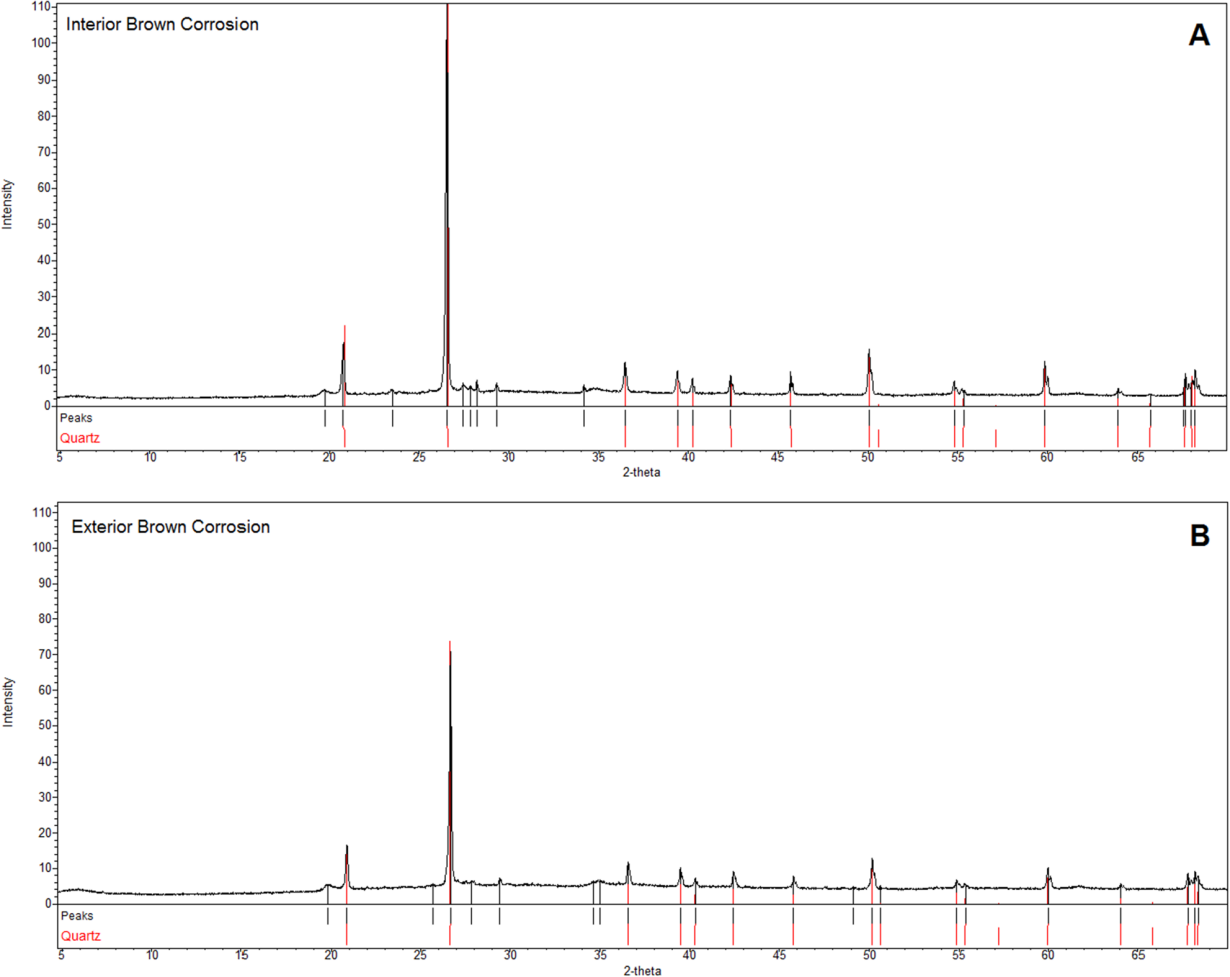
Diffractogram from the XRD analysis of the brown corrosion material from interior of the bowl (**A**) and exterior (**B**) showing the identified mineral phase.

The only mineral phase identified in the brown corrosion samples (Fig. 3) was quartz.

### Spectroscopy analysis found evidence for chlorides and aromatic compounds in green corrosion

**Figure 4:**
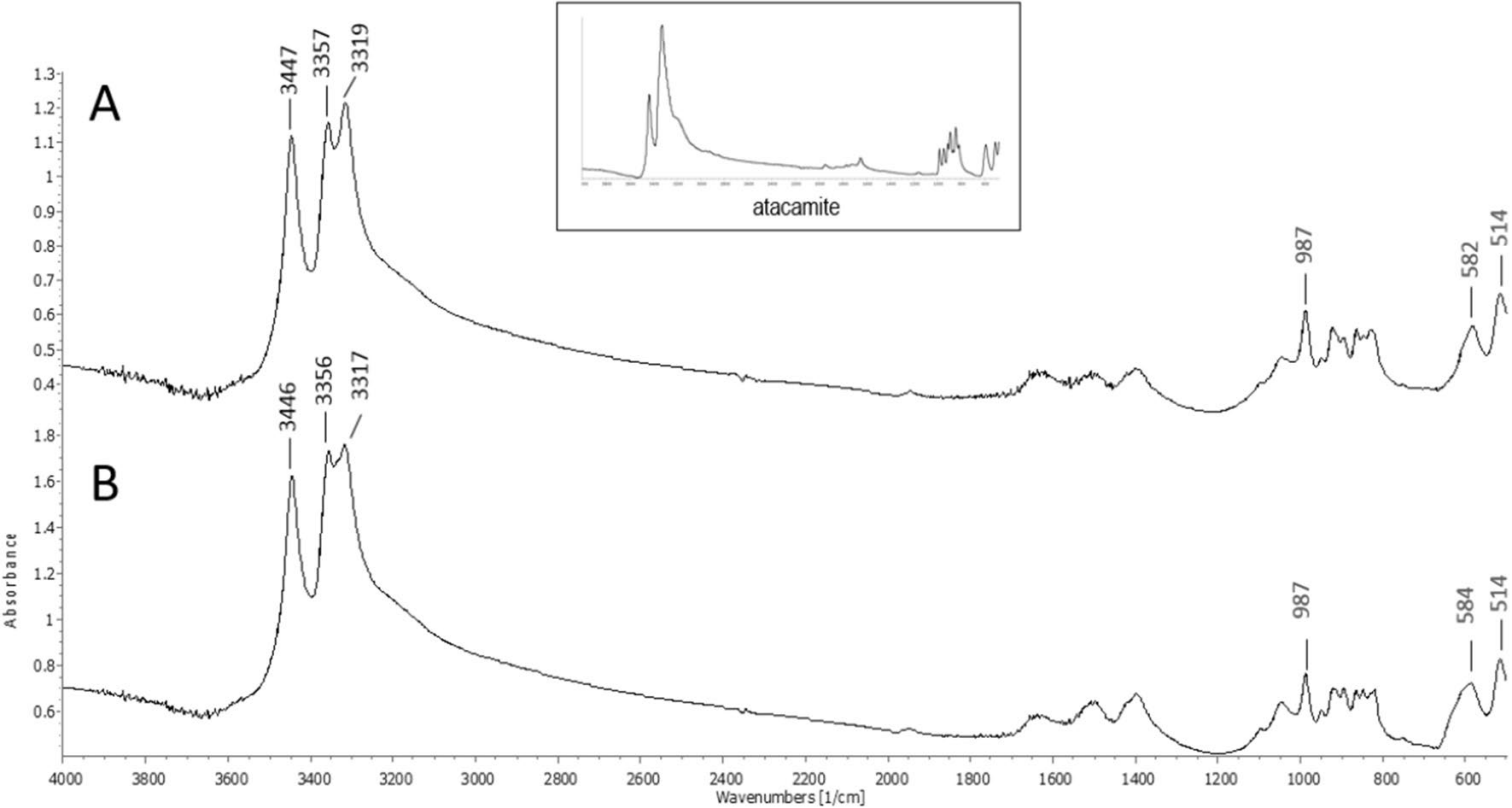
FTIR spectra for green corrosion from the interior (**A**) and exterior (**B**). Peaks in red also found in atacamite’s spectrum (inset).

The FTIR spectra obtained for the green corrosion samples have poorly resolved bands and are practically identical (Fig. 4). The most intense bands appear at higher frequencies at around 3447, 3356 and 3317 cm^−1^ and are associated to O–H and/or N–H stretching vibrations. In the fingerprint region (i.e. below 1500 cm^−1^) bands are visible around 987, 582 and 514 cm^−1^. Some of these bands may result from the contribution of atacamite’s spectrum (Fig. 4 inset).

The broad band between 1600 and 1400 cm^−1^ may indicate the presence of an aromatic ring system, which normally appears as four bands. The absence of strong bands at 2900–2880 cm^−1^ indicates that waxes, resins or oils are either absent or present at quantities below the detection limit of the technique.

There was little difference between the spectra of the brown corrosion samples (included in the Supplementary Information Figure S1). Both spectra contained poorly defined peaks: a broad band centred around 3400 cm^-1^ due to O–H/N–H stretching vibrations and a strong broad peak (better defined in the spectrum of the exterior sample) at around 1050 cm^-1^ for Si–O stretching in silicates and/or C–O stretching in polysaccharides^6^. As in the green corrosion samples, no peaks were visible in the 2900–2880 cm^-1^ region.

### Chlorinated aromatic compounds identified in the green corrosion

**Figure 5:**
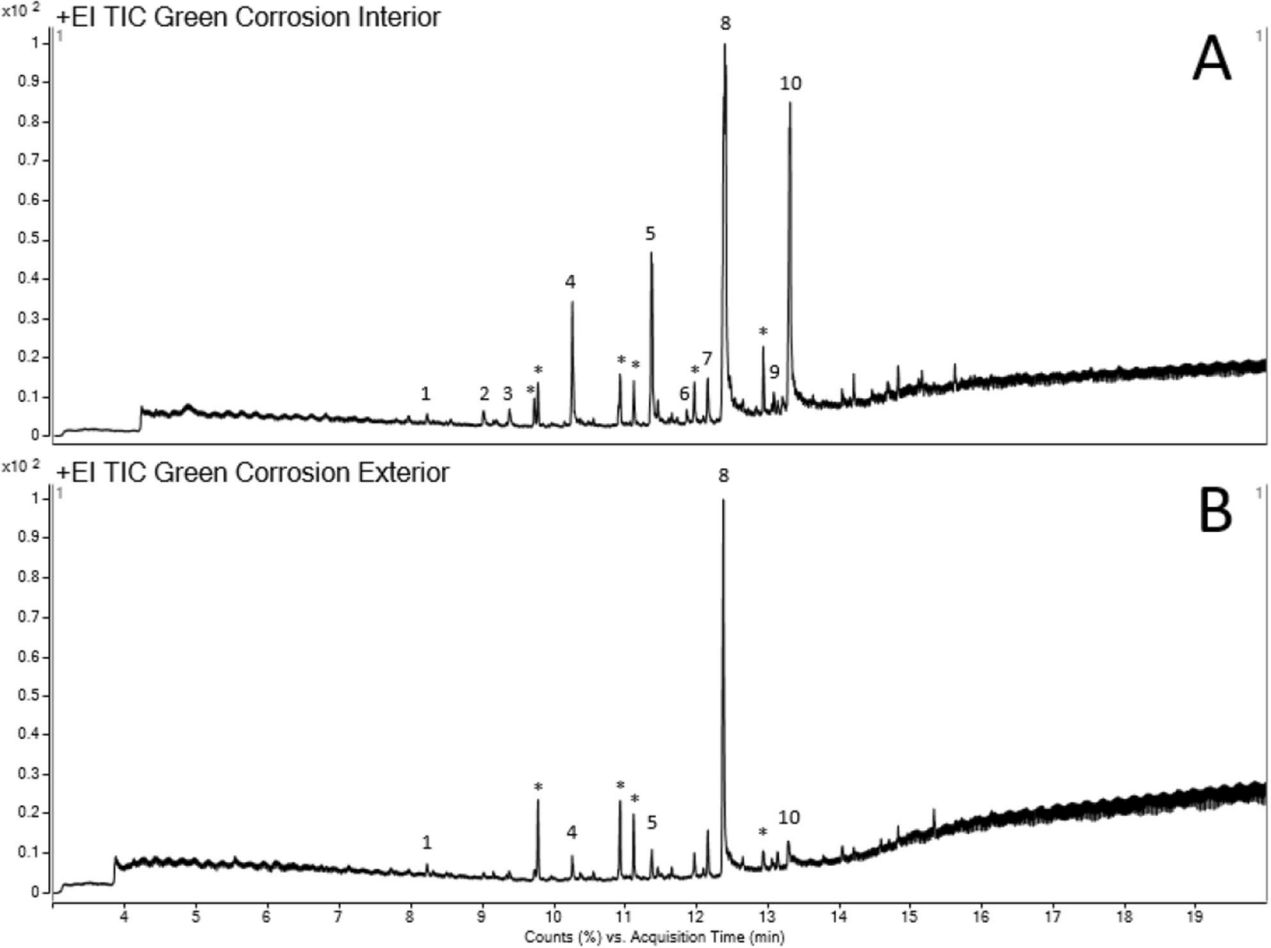
GC–MS mass chromatograms from the analysis of green corrosion material from the interior (**A**) and exterior (**B**) of the excavated bowl. Compounds identified by accurate mass and fragmentation pattern matching with NIST library labelled as: [1] 1,2,3-trichlorobenzene; [2] 1,2,4,5-tetrachlorobenzene; [3] 1,2,3,5-tetrachlorobenzene [4]; pentachlorobenzene; [5] hexachlorobenzene; [6] pentadecanoic acid; [7] hexadecanoic acid, methyl ester; [8] n-hexadecanoic acid; [9] methyl stearate; [10] n-octadecanoic acid; [*] non-specific compounds from plant material. Spectra associated with chlorobenzenes’ assignments included in Supplementary Figs. S2-6.

Mass chromatograms obtained from the analysis of the green corrosion material by GC-QTOF-MS with TSP (Fig. 5) contain high intensity peaks identified as hexadecanoic acid [8] and chlorobenzenes [1, 2, 4 and 5]. Chlorobenzenes are synthetic compounds used in the production of organic chemicals (as solvents), in deodorants, fumigants, herbicides and as pesticides^7^. The mass chromatogram obtained from the analysis of the interior sample (Fig. 5A) also contains a major peak for octadecanoic acid [10], which appears at a lower intensity in the chromatogram of the exterior sample (Fig. 5B). Pentadecanoic acid [6] and methyl esters [7 and 9] were only identified in the interior sample, with unassigned peaks for long-chain alkanes, alkenes and alkanols, possibly of plant origin, present in both samples.

**Figure 6:**
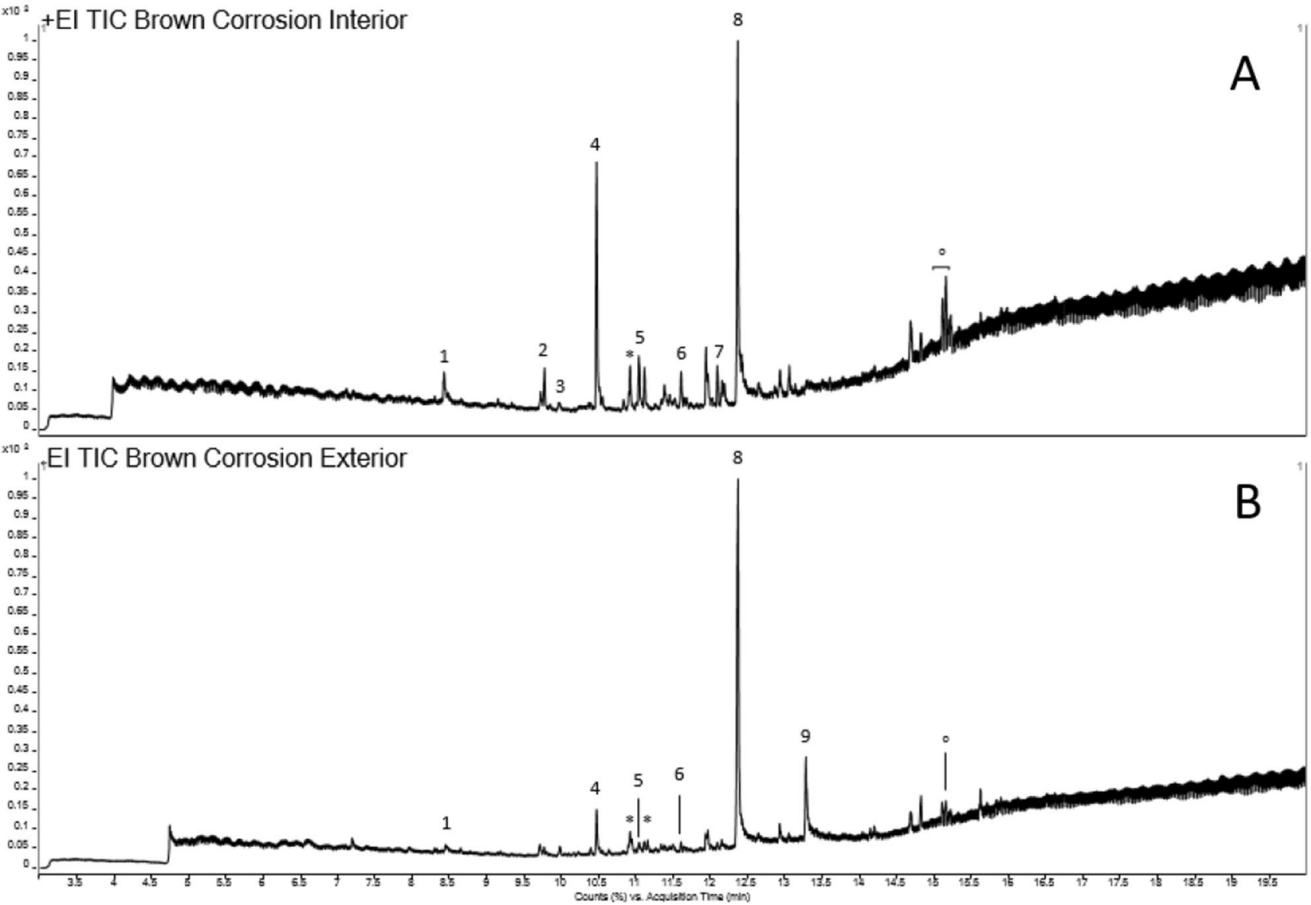
GC–MS mass chromatograms from the analysis of brown corrosion material from the interior (**A**) and exterior (**B**) of the excavated bowl. Compounds identified by accurate mass and fragmentation pattern matching with NIST library labelled as: [1] Methenamine; [2] Ceteneo; [3] 2,4-Di-tert-butylphenol; [4] Diethyltoluamide (DEET); [5] N-ethyl-2-methyl-benzenesulfonamide,; [6] 2-Propanol, 1-chloro-,phosphate (3:1); [7] Hexadecanenitrile; [8] n-hexadecanoic acid; [9] octadecanoic acid; [*] plant material; [^o^] polycyclic compounds. Spectra associated with DEET assignment included in Supplementary Fig. S7.

The mass chromatogram obtained from the analysis of the brown corrosion from the interior of the bowl (Fig. 6A) is dominated by two peaks: hexadecenoic acid [8] and diethyltoluamide (DEET) [4], its accurate mass and fragmentation pattern matching is included in Supplementary Figure S7. DEET was developed by the United States Army in 1946^8^, and is the active ingredient in many insect-repellent products for topical use in humans and livestock^9^. There is also evidence of DEET in the exterior sample (peak 4 in Fig. 6B), where the second most prominent peak was identified as octadecanoic acid [9]. Amongst the unidentified compounds in both samples are possible compounds of plant origin and polycyclic substances, which could not be assigned to a specific compound.

## Discussion

### Factors affecting corrosion of the bowl

The most important parameters governing the corrosion of copper-alloy objects in soils are moisture, salt content, temperature, acidity and aeration^10–13^. The geology of the site where the bowl was found is generally described as boulder clay over chalk bedrock^14,15^, freely drained and slightly acidic^16^. According to short-term corrosion studies, copper and its alloys are expected to develop only sporadic localized pit corrosion in this type of soil^17^. However, it is expected that the chemical characteristics of the soil where the Roman bowl was found would have been affected by human agency over more than a thousand years during the time the object had been buried. A Swedish study^13,18^ found that recently-excavated bronzes were more extensively corroded compared to other bronze objects from the same site in museum collections. The researchers attributed the worst state of preservation of recently-excavated metal objects to an increase in soil acidity due to anthropogenic pollution in the last 50–100 years.

The site where the bowl was found has been used for agriculture since at least 1936 and, in the last ten-twenty years, planted as an orchard. Hexachlorobenzene (HCB), tri, tetra and penta-chlorobenzenes were used in agriculture as fungicides, herbicides, insecticides and pesticides^19^. In soils, highly substituted chlorobenzenes degrade via a range of reductive de-chlorination reactions^20,21^, essentially. Consisting of cleavage of the C–Cl bond following an electron transfer^22,23^, often by microbial action under anaerobic conditions^24,25^. Chlorobenzenes of lower substitution number are less toxic and more susceptible to microbial degradation^26–28^, with eventual mineralization into carbon dioxide, water and chloride ions^29,30^. The presence of metals^31^, including copper^32–35^, catalyse the degradation of HCB, with the opposite effect observed when co-existing chlorobenzenes are present under a neutral to alkaline pH^36,37^.

The identification of chlorobenzenes and basic copper chlorides in the bowl’s green corrosion suggests a relationship between these compounds because the soil where the object was found is characterized by a low concentration of chloride ions^38^. The heterogeneity of metallurgical features of the copper alloy^39,40^ used to produce the bowl and damage to the protective copper oxide layer would have supported the formation of anodic pits^41^. Anodic pits are positively-charged areas that are likely to have attracted chloride ions from the degradation of chlorobenzenes, leading to the formation of basic copper chlorides. This corrosion mechanism is supported by the fact that neither chlorobenzenes nor copper corrosion mineral phases were identified in the brown samples (composed by quartz mixed with non-chlorinated organic compounds).

Archaeological evidence for the effect of agrochemicals on the corrosion of buried copper alloy objects is limited to the effect of fertilizers^42^. In 2004, a group of scientists attempted to gather empirical evidence that agrochemicals accelerate the corrosion of metal objects buried in the soil^43^ but the difficulty in corroding copper in soil had a negative impact in their laboratory experiments^44^. Notwithstanding these difficulties, the researchers’ geochemical modelling predicts the following order of corrosiveness with respect to inorganic fertilisers: KCl (muriate of potash) > nitrogen > phosphorus^45^. The K-Cl bond disassociation energy is 427 kJ/mol while for C–Cl bond in thirteen chlorobenzenes range between 375 to 399 kJ/mol^46^, making chlorobenzenes more corrosive than KCl-based fertilizers.

### Sources and fate of chlorobenzenes

Chlorobenzenes are synthetic compounds formed by the addition of up to 6 chlorine atoms to a benzene ring. Given the low solubility of chlorobenzenes in water (which is inversely proportional to the number of chlorine atoms), they are relatively resistant to chemical degradation. HCB is the most unreactive and well-studied chlorobenzene^47,48^. It was introduced as an agricultural pesticide in 1945^49^, with emissions peaking in mid-1960s. Given its toxicity to humans^50,51^, and ability to accumulate in the environment and living organisms^52^, HCB is considered a persistent pollutant and its in agriculture is now restricted in most countries^53^.

Although HCB has been banned in the UK since 1975, analyses of 1968–1990 archived rural soils did not reveal a pronounced decline in HCB detected in soil samples collected since restrictions were introduced^54^. It is possible these results also include contributions from secondary sources such as industrial emissions, application of pesticides containing HCB^51^ such as dimethyl tetrachloroterephthalate (DCPA, traded as Dacthal), pentachloronitrobenzene (sold as PCNB Terraclor, Engage, and Defend), hexachlorocyclohexane (trade name Technical HCB or Mirex), pentachlorophenol and Picloram)^55^, sewage sludge^56^ and irrigation with contaminated water. The complexity of environmental contamination by persistent pollutants is exemplified by Diethyltoluamide (DEET)^57^ detected in the bowl’s brown corrosion. DEET is mainly associated with insect-repellent of personal use and an emerging pollutant in England’s groundwater systems, with the highest abundance found in natural settings^58^.

### Potential policy implications

In the UK, the arrival of the twentieth century marked the development of archaeology as a professional discipline^59^. Bombing of cities during the Second World War exposed many archaeological sites, with the creation of many Planning Acts to control the redevelopment of these sites and funds allocated to support excavations. English Heritage was created in 1983 to advise the government on heritage matters, with similar institutions created in Wales, Scotland and Northern Ireland. Initially English Heritage had its own field excavation unit, which was eventually closed in 1990. This closure coincided with the adoption of Planning Policy Guidance Note 16 (PPG16), which made archaeology a material factor to be considered before determining planning applications for the redevelopment of sites. PPG16 states that when construction works would disturb the archaeological remains present in a site, the developer may be obliged to excavate, record and publish those assets. The adoption of PPG16 and subsequent legislation secured access to sites and funding for archaeological excavations, creating an unprecedented demand for commercial archaeological services^60^. By 2007, 93% of all archaeological excavations in the UK were developer-led^61^.

Under this legal framework, it is implied that archaeological remains buried underground are largely protected from damage, particularly in agricultural land if buried below the topsoil (and thus protected from mechanical disturbances). In the case of metal objects, it is presumed that corrosion rates would have slowed down since initial deposition, with chemical reactions reaching an equilibrium with the burial environment. Excavation exposes these objects to different environmental conditions from those in the deposition environment such as an increase in oxygen levels and humidity which can promote further corrosion processes.

A small number of studies have been published linking corrosion of archaeological metal objects to agricultural activities^42,62^ but these have been limited to the identification of “unusual” mineral phases. However, our study indicates that archaeological copper alloy objects provide a sink for chlorobenzenes, producing more familiar diagnostic chemistry (i.e. atacamite and paratacamite), therefore presenting an opportunity to systematically evaluate corrosion products from freshly-excavated objects. Such evaluation would provide a clearer picture of the impact of soil pollutants on archaeological objects and the means to monitor their distribution.

In the UK metal detectorists are responsible for most of the metal finds coming out of rural lands, which are reported under the Portable Antiquities Scheme (PAS)^63^. Corrosion samples could be submitted for characterization through the PAS platform enabling correlation of results with historical records of land use and soil environmental surveys. Such an initiative would provide metal detectorists, who are often marginalized by heritage professionals^64^, an opportunity to contribute to cultural and environmental heritage management policies. Publishing material to raise awareness about the effect of pollutants on copper alloy objects amongst this community and mechanism for reporting it would be the first step to facilitate engagement.

## Conclusion

We found evidence of chlorobenzenes amidst corrosion from an archaeological copper alloy bowl excavated from a rural site. Chlorobenzenes are synthetic compounds released in the environment through agricultural and industrial activities. Given their toxicity and persistence in the environment, many countries have taken steps to control their release. Our study provides the first evidence that chlorobenzenes are associated with accelerated corrosion mechanisms linked to archaeological material and demonstrates that they are a threat to the preservation of archaeological metals in the ground.

## Methods

### Fourier-transform infrared (FTIR)

Samples were analysed in transmittance mode using the KBr pellet method in an Excalibur Series Varian UMA600. Measurements were taken between the 4000-400 cm^−1^ range, 64 scans and included background subtraction. Data was processed using Digilab Resolutions Pro 4.0 software and figures created with Spectragryph v.1.2.12.

### Gas chromatography quadrupole time-of-flight mass spectrometry using a thermal separation probe (GC-QTOF-MS with TSP)

Around 4 mg of sample ground to a powder in an agate mortar was transferred to a glass microvial and placed inside the TSP attached to an Agilent 7890B gas chromatograph equipped with a Restek Rxi-5 ms column (30 m × 320 μm × 0.25 μm) attached to an Agilent 7250 GC/QTOF mass spectrometer equipped with a low-energy-capable electron ionization source operating at 70 eV. The TSP was set at 300 °C. The oven temperature was set at 40 °C for one minute, increasing by 20 °C/minute until 320 °C, where it was held for five minutes. Helium was used as a carrier gas, at 1.43 mL/min flow rate and 8.70 psi pressure. The equilibration time was set at 0.5 min and the sample injection was splitless. The mass range was 50 to 650* m/z*. All samples were run in triplicate.

Data analysis was performed with Agilent Mass Hunter Qualitative Analysis 10.0 with compound assignments using NIST Library 17. Only compounds with a match factor (MF) and reverse match factor (RMF) > 700 and accuracy mass error below 50 ppm were shortlisted.

### X-ray powder diffraction (XRD)

The sample was pulverized and set on a silicon plate and analysed using a PANalytical X'Pert PRO Cu alpha instrument set to operate in continuous mode at 40 kV/40 mA with a scanned area set between 1 and 70° 2θ, 0.02 step size and 3° per minute.

Data was processed using QualX© software with phase identification using the Crystallography Open Database (COD).

## Supplementary Information

Supplementary Information.

## Data Availability

The raw datasets are available from the corresponding author on reasonable request.
