# Supplementary material for: The influence of pesticides on the corrosion of a Roman bowl excavated in Kent, UK
Source: Sci Rep. 2022 Oct 6;12:14521. doi: 10.1038/s41598-022-17902-9 (PMC9537325; doi:10.1038/s41598-022-17902-9)
Supplement: Supplementary file 1 — Supplementary Information. [file 41598_2022_17902_MOESM1_ESM.docx]

**The influence of pesticides on the corrosion of a Roman bowl excavated in Kent, UK**

Luciana da Costa Carvalho^1,2^*, Dana Goodburn-Brown^2^, James S. O. McCullagh^3^ and A. Mark Pollard^1^

^1^ School of Archaeology, University of Oxford, Oxford OX1 3TG, UK

^2^CSI: Sittingbourne, 22 The Forum, Sittingbourne ME10 3DL

^3^Chemistry Research Laboratory, Department of Chemistry, University of Oxford, Oxford OX1 3TA, UK

**Supplementary Information**

[**Supplementary Figure S1** - FTIR spectra for brown corrosion from the interior (A) and exterior (B) 2](#_Toc111207853)

[**Supplementary Figure S2 – [1] 1,2,3-trichloro benzene** 3](#_Toc111207854)

[**Supplementary Figure S3 - [2] 1,2,4,5-tetrachloro benzene** 4](#_Toc111207855)

[**Supplementary Figure S4 - [3] 1,2,3,5-tetrachloro benzene** 5](#_Toc111207856)

[**Supplementary Figure S5 – [4] pentachloro-benzene** 6](#_Toc111207857)

[**Supplementary Figure S6 - [5] hexachloro-benzene** 7](#_Toc111207858)

[**Supplementary Figure S7 - [4] Diethyltoluamide (DEET)** 8](#_Toc111207859)

**
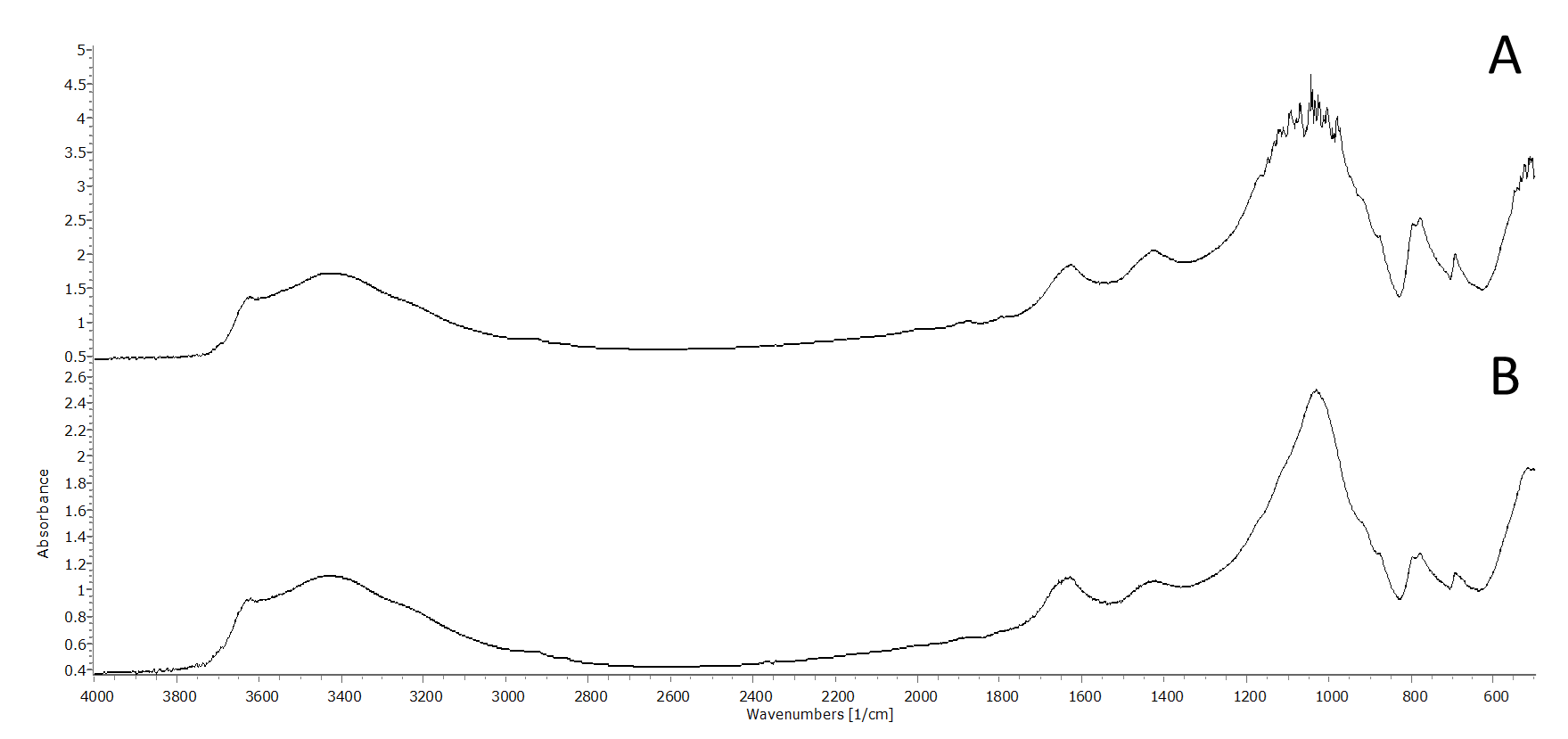
**

**Supplementary Figure S1** - FTIR spectra for brown corrosion from the interior (A) and exterior (B)

**NIST Identification spectra**

| **B**  **A**  ****  MF=862 RMF=893  Measured *m/z*=179.9297  mass error = -1.85ppm | 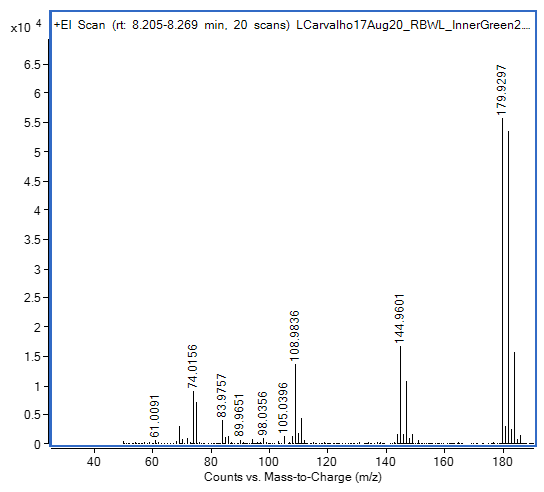 |
| --- | --- |
| ****  MF=880 RMF=911  Measured *m/z*=179.9283  mass error = -9.63ppm | 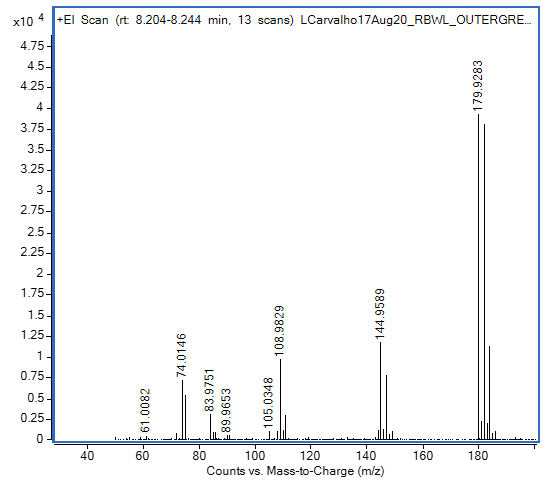 |

Key: green corrosion from the interior (A) and exterior (B)

**Supplementary Figure S2 – [1] 1,2,3-trichloro benzene**

**NIST Identification**

MF=854 RMF=900

Measured *m/z*=213.8894

mass error = -7.77ppm

**spectrum**


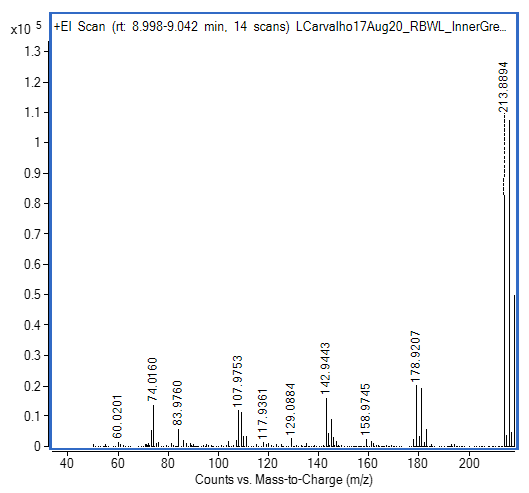


**Supplementary Figure S3 - [2] 1,2,4,5-tetrachloro benzene**

**NIST Identification**

**
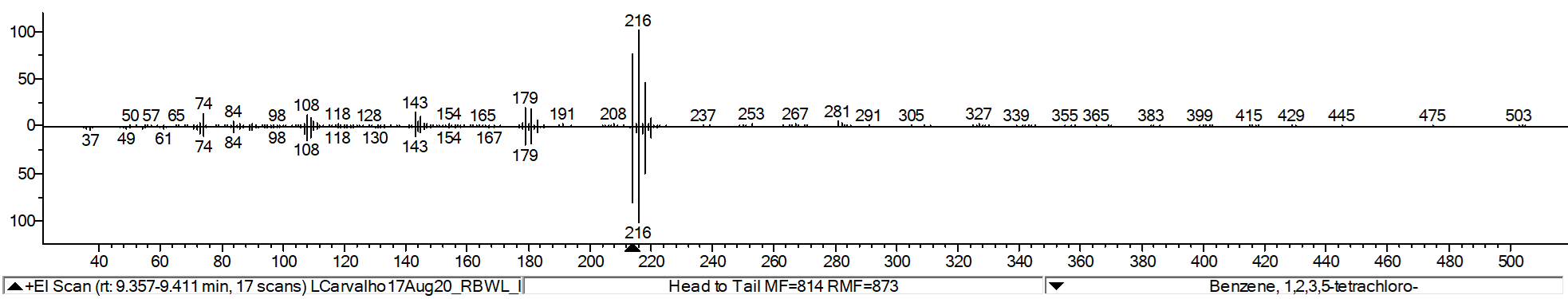
**

MF=814 RMF=873

Measured *m/z*=213.8889

mass error = -10.10ppm

**spectrum**


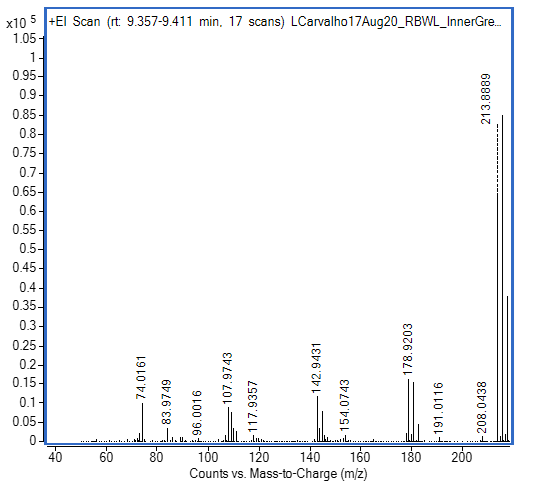


**Supplementary Figure S4 - [3] 1,2,3,5-tetrachloro benzene**

**NIST Identification spectra**

| **B**  **A**  **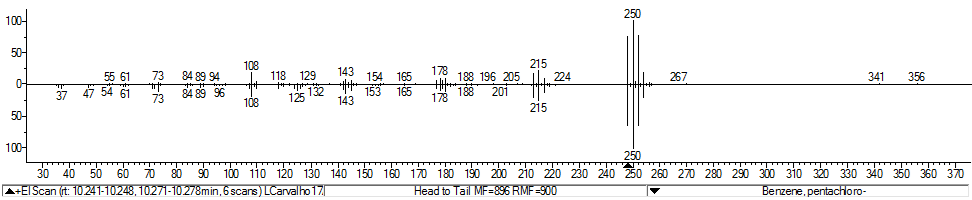**  MF=896 RMF=900  Measured *m/z*= 247.8543  mass error = 8.92ppm | **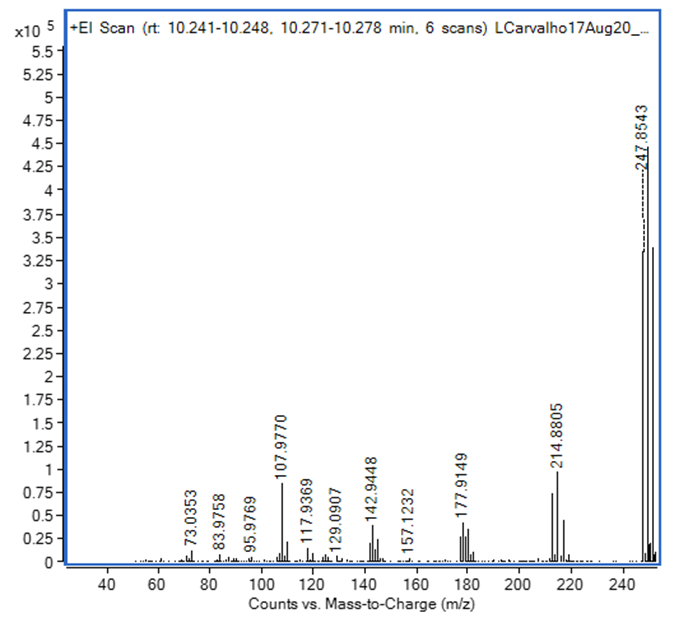** |
| --- | --- |
| **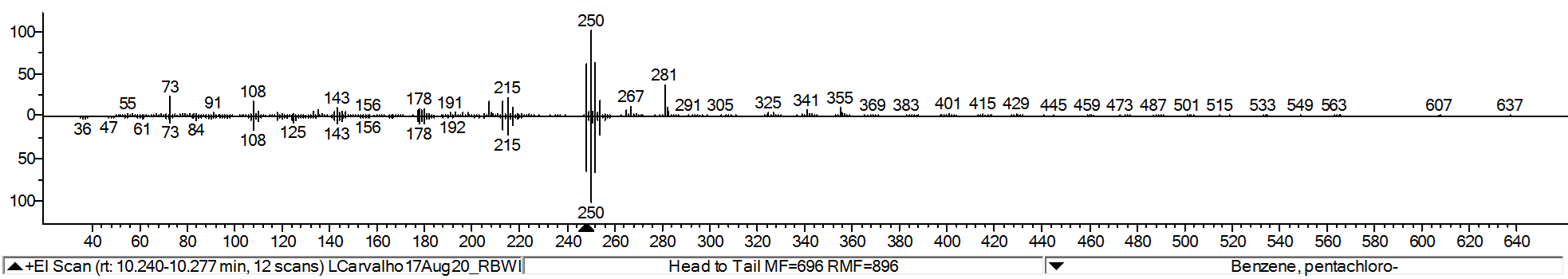**  MF=696 RMF=896  Measured *m/z*= 247.8500  mass error = -8.42ppm | 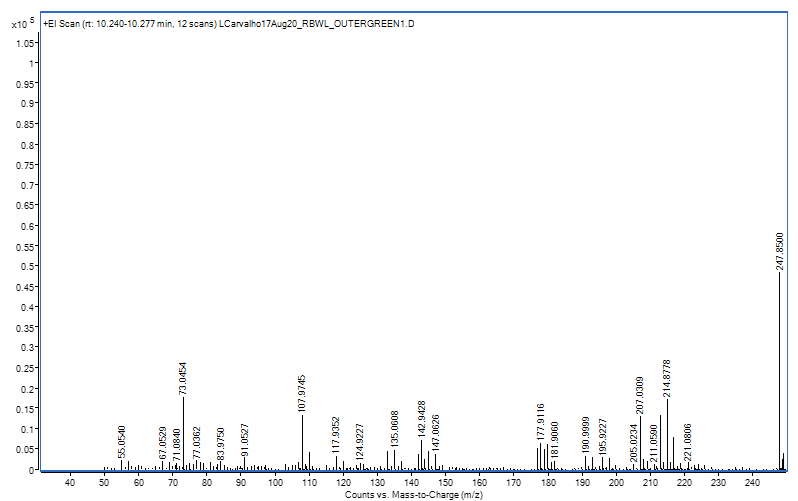 |

Key: green corrosion from the interior (A) and exterior (B)

**Supplementary Figure S5 – [4] pentachloro-benzene**

**NIST Identification spectra**

| **B**  **A**  **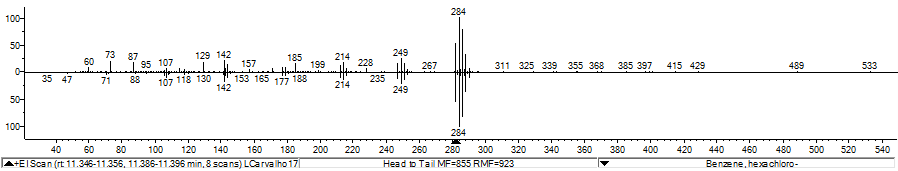**  MF=855 RMF=923  Measured *m/z*= 281.8101  mass error = -10.70ppm | **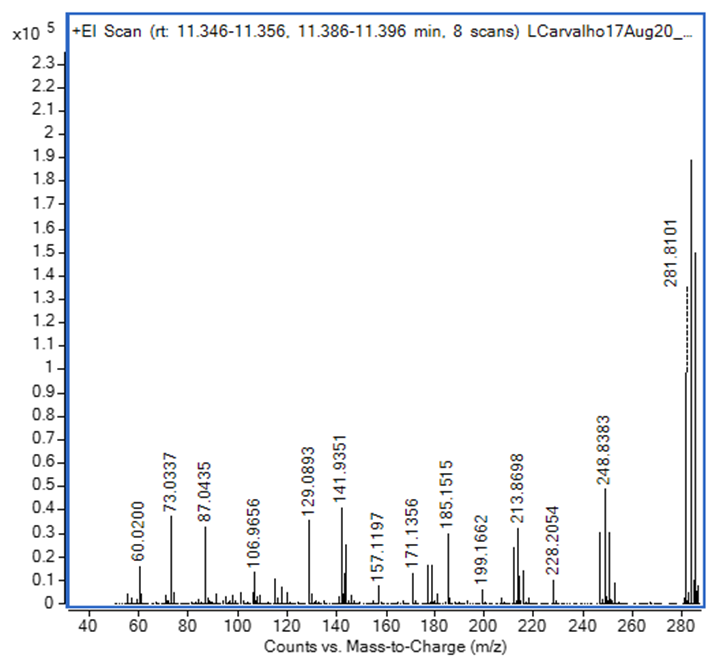** |
| --- | --- |
| **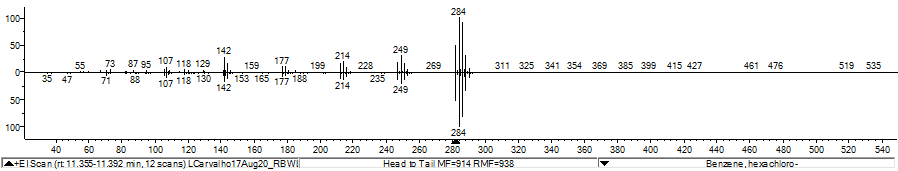**  MF=914 RMF=938  Measured *m/z*= 281.8114  mass error = -6.09ppm | **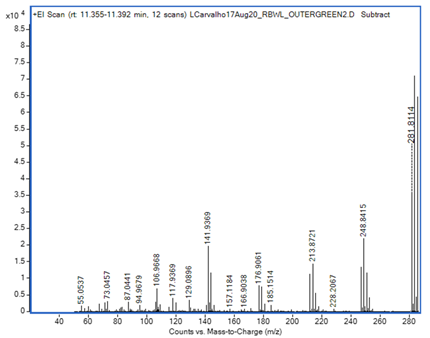** |

Key: green corrosion from the interior (A) and exterior (B)

**Supplementary Figure S6 - [5] hexachloro-benzene**

**NIST Identification spectra**

| **B**  **A**  ****  MF=914 RMF=919  Measured *m/z*= 191.1293  mass error = -8.97ppm | 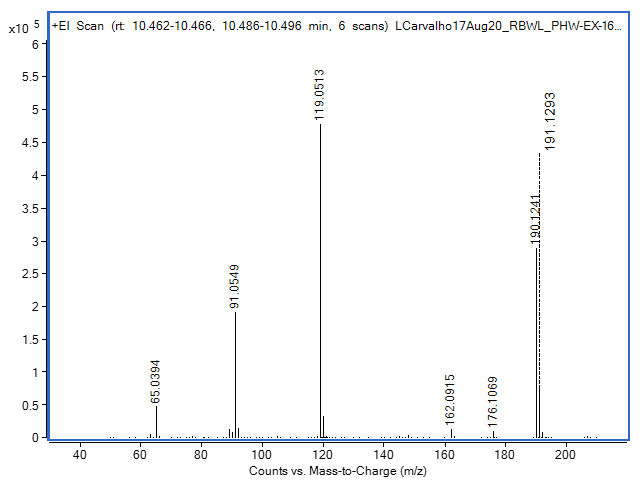 |
| --- | --- |
| ****  MF=849 RMF=891  Measured *m/z*= 191.1242  mass error = -35.65ppm | 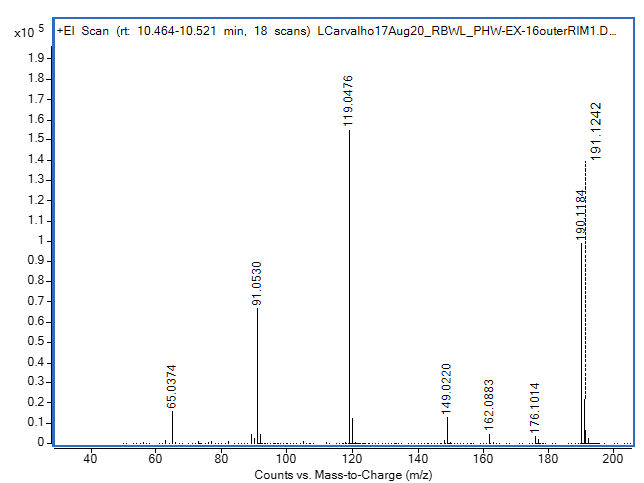 |

Key: brown corrosion from the interior (A) and exterior (B)

**Supplementary Figure S7 - [4] Diethyltoluamide (DEET)**
